# Heat Shock Proteins in Head and Neck Squamous Cell Carcinoma

**DOI:** 10.3390/cells14231897

**Published:** 2025-11-28

**Authors:** Piotr Cierpikowski, Julia Bar

**Affiliations:** 1 Department of Maxillofacial Surgery, The Ludwik Rydygier Specialist Hospital, Osiedle Zlotej Jesieni 1, 31-826 Krakow, Poland; 2 Department of Immunopathology and Molecular Biology, Wroclaw Medical University, Borowska 211, 50-556 Wroclaw, Poland

**Keywords:** heat shock proteins, head and neck cancer, molecular targeted therapy

## Abstract

Heat shock proteins (HSPs) are produced in response to stressful conditions, such as temperature, inflammation, infection, or exposure to environmental factors. HSPs are overexpressed in some malignancies, where they modulate the tumor microenvironment and influence cancer cell behavior and survival. Clinical trials for breast, prostate, colon, and lung cancers exist, but not for head and neck squamous cell carcinomas (HNSCCs). Nonetheless, clinical studies on HSPs in HNSCC are still lacking. We review the role of HSPs with regard to physiology and as potential targets for molecular therapy in HNSCC.

## 1. Introduction

Heat shock proteins (HSPs) are a family of molecular chaperones that play a role in protein synthesis, secretion, folding, stabilization, translocation, and proteolysis [1,2]. Although the precise role of HSPs in tumor biology is unclear, HSPs appear to be involved in carcinogenesis and drug resistance [2,3,4].

This paper is a summary of the current knowledge on HSPs (especially HSP27, HSP70, and HSP90) in head and neck squamous cell carcinoma (HNSCC). We also consider the potential role of HSPs in their pathogenesis and the possible use of HSP inhibitors in therapy.

## 2. The HSP Family: Characteristics and Classification of HSPs

HSPs play a regulatory role as chaperones in protein-folding homeostasis and are involved in proliferation, differentiation, apoptosis, and responses to stress [3,5,6,7,8]. HSPs are activated by stressors such as high temperature, hypoxia, lack of nutrients, ischemia, infection, and inflammation [2,4]. They prevent protein aggregation by binding to misfolded proteins and helping them refold when the stress subsides [2]. HSPs prevent the accumulation of damaged proteins by regulating the ubiquitin–proteasome system, which is involved in the degradation of aggregated or improperly folded proteins [4]. HSPs constitute 5–10% of all proteins in normal cells and increase in response to stress [4,9]. They range in size from 10 kDa to >100 kDa and are divided into small HSPs [sHSPs—HSP20 and HSP27], HSP40, HSP60, HSP70, and HSP90, and large HSPs [HSP105 and HSP110] [4,10,11].

Ten sHSPs [HSPB1 to HSPB10] are first responders to cellular stress and do not use ATP [3,4,6,12]. Their α-crystallin domain (ACD) sequence is located near the C-terminus [11]. HSPB1, also called HSP27, is its most prominent member [6].

The HSP40 family is divided into three subclasses: Type I (DNAJA), Type II (DNAJB), and Type III (DNAJC) [8]. They have a highly conserved J-domain composed of four α-helices. They may interact with HSP70 by stimulating ATPase activity [6,8]. HSP40 assists in processes concerned with folding, unfolding, translocation, and protein degradation [6].

HSP60 (also known as chaperonin or Cpn60) is highly conserved [4]. Structurally, it comprises a double ring shape with three domains: equatorial, intermediate, and apical [13]. Its activity is controlled by the binding of ATP to the equatorial domain [8]. It is responsible for the transport and folding of mitochondrial proteins [14] and regulating apoptosis in response to stressors [13].

HSP70, a large family of ATP-dependent, highly conserved proteins, has two main domains: the nucleotide-binding domain (NBD) and the substrate-binding domain (SBD) [4,15]. NBD binds to and hydrolyzes ATP, thereby regulating its activity [8]. The SBD interacts with client proteins, assisting in folding and preventing aggregation [8]. HSP70 activity is controlled by the J-domain protein (JDP) and nucleotide exchange factor (NEF) [16].

HSP90 is the most-studied HSP, which regulates many signaling pathways involved in intracellular protein stability [17]. It is highly conserved, ATP-dependent, and expressed by six genes in humans [4,8]. It has three domains: C-terminal (CTD), middle (MD), and N-terminal nucleotide-binding (NTD) [4,8]. Its activity is based on ATP-dependent conformation changes that may be regulated by binding co-chaperones such as HSP40, HSP70, p23, Csc37, Aha1, Hip, and Hop [8].

The large HSPs (including HSP105, HSP110, and Grp170) are a subfamily that prevents substrate aggregation [4]. As they are homologous to the HSP70 family, they are often described as the “HSP70 superfamily” [4]. HSP110 serves as a co-chaperone for HSP70 to improve its folding capability [8].

## 3. The Role of HSPs in Cancer

**Figure 1:**
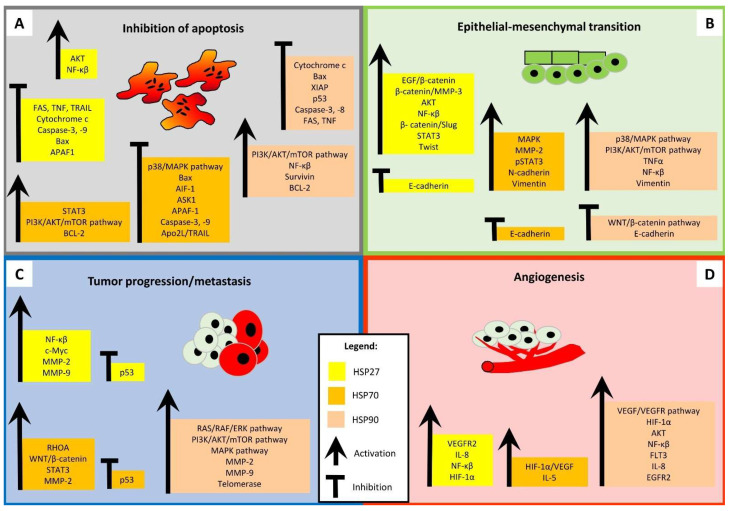
The cellular processes mediated by HSP27, HSP70, and HSP90 proteins in carcinoma. (**A**) **Inhibition of apoptosis:** HSP27 inhibits apoptosis by interacting with AKT, activating NF-κβ, blocking Bax, cytochrome c, caspase-3, caspase-9, and suppressing FAS, TNF, and TRAIL receptors. HSP70 blocks apoptosis by the activation of the STAT3, BCL-2, and PI3K/AKT/mTOR pathways and suppression of the p38/MAPK pathway, Bax, AIF-1, ASK1, APAF-1 proteins, caspase-3, caspase-9, and Apo-2L/TRAIL. HSP90 inhibits apoptosis by the activation of the PI3K/AKT/mTOR pathway, NF-қβ, survivin, BCL-2 and the suppression of cytochrome c, Bax, XIAP, p53, caspase-3, caspase-8, FAS, and TNF receptors. (**B**) **Epithelial–mesenchymal transition (EMT):** HSP27 mediates EMT by the activation of EGF/β-catenin, β-catenin/MMP-3, β-catenin/Slug, NF-κβ, AKT, STAT3, and Twist and the decrease in E-cadherin expression. HSP70 regulates EMT by the activation of MAPK, MMP-2, pSTAT3, N-cadherin, vimentin and downregulation of E-cadherin. HSP90 controls EMT by the activation of the p38/MAPK and PI3K/AKT/mTOR signaling pathways, TNFα, NF-қβ, vimentin, and the inhibition of WNT/β-catenin pathways, and E-cadherin expression. (**C**) **Tumor progression/metastasis:** HSP27 promotes tumor progression and the formation of metastasis by the activation of NF-қβ, c-Myc, MMP-2, and MMP-9 and the downregulation of the p53 protein. HSP70 influences tumor progression and formation of metastasis by the activation of RHOA, WNT/β-catenin pathway, STAT3, and MMP-2 and the downregulation of p53 expression. HSP90 effects tumor progression and formation of metastasis by the activation of the RAS/RAF/ERK, PI3K/AKT/mTOR, and MAPK signaling pathways, MMP-2, MMP-9, and telomerase upregulation. (**D**) **Angiogenesis:** HSP27 increases angiogenesis by the upregulation of VEGFR2, IL-8, NF-қβ, and HIF-1α. HSP70 promotes angiogenesis by the upregulation of HIF-1α/VEGF activity and IL-5 secretion. HSP90 increases angiogenesis by the activation of HIF-1α, NF-қβ, AKT, VEGF/VEGFR signaling pathway and the upregulation of FLT3, IL-8, and EGFR2.

Dysregulated HSP expression is associated with several diseases, including cancer [1,18,19]. It is generally accepted that HSPs play a key role in tumor initiation and progression (Figure 1). Dysregulation of the chaperonage machinery inhibits the targeting of oncoproteins and signaling pathways participating in tumor development and progression [6,18,19,20,21]. Overexpression of HSPs induces tumorigenesis in HNSCC (or cell lines) involved in carcinogenesis, epithelial–mesenchymal transition (EMT), metastasis, and resistance to irradiation and chemotherapy [3,5,20,22,23]. This review focuses on HSP27, HSP70, and HPS90 as there is less information available for other HSPs. These three HSPs are overexpressed or upregulated in tumors, but their specific roles and correlations with other proteins are not well defined [24].

### 3.1. HSP27

HSP27 plays a key role in cell proliferation, invasion, metastasis, and apoptosis. It is expressed in normal cells at basal levels. Conformational changes are induced by phosphorylation at one of the specific sites (Serine 15, Serine 78, Serine 82, or Threonine 143) through MAPKAP kinase 2/3 [6]. The phosphorylation status is closely related to cancer disease [1,25]. HSP27 levels increase, and there is a different function than in normal cells [26]. HSP27 interacts with different oncoproteins, receptors, and signaling pathway components (Figure 1). Its overexpression reduces cell death by apoptosis in response to various stressful factors such as hypoxia and cytotoxic drugs.

Phosphorylated HSP27 participates in extrinsic and intrinsic pathways of apoptosis, suppresses caspase-3 activity, and inactivates Bax and Daxx proteins [4,27]. HSP27 blocks caspase activity by blocking the release of SMAC Diablo and cytochrome c from mitochondria, resulting in a lack of interaction between cytochrome c and apoptotic protease-activating factor-1 (APAF-1) and procaspase-9 [1,4,6]. Phosphorylation of AKT by HSP27 prevents apoptosome formation and induces cell survival through Bax inhibition [6]. HSP27 also suppresses other apoptosis pathways such as FAS, TNF, and TRAIL [1,4,27]. HSP27 may regulate cellular senescence by HDM2 destabilization and p53 stabilization [6]. It may also inhibit several p53-related functions [6,18], such as p21 protein-inhibiting senescence in response to p53 activator nutlin-3.

HSP27 is involved in EMT, which allows epithelial cells to leave their polarized organization and intercellular adhesion, and to increase tumor invasiveness and migratory properties [4,28,29]. HSP27 triggers the phosphorylation of STAT3, which then binds to the Twist promoter and enhances EMT [30]. HSP27 overexpression might result in epidermal growth factor (EGF) signaling pathway/β-catenin activity: β-catenin binding to the SNAIL 2 (Slug) promoter, increasing metallopeptidases (MMPs) [4,26,28]. HSP27 induces the suppression of E-cadherin with concurrent activation of other EMT markers, such as vimentin, Slug, and fibronectin [4,28]. HSP27 influences EMT by c-Myc and p53 protein loss [31,32].

HSP27 may suppress apoptosis in oral cancer and facilitate tumor immune rejection by modulating the β-catenin/MMP-3 signaling cascade and by the upregulation of NF-ĸB signaling pathways [27,31,33]. HSP27 involvement in oral squamous cell carcinoma (OSCC) is unclear: it is overexpressed in dysplasia and OSCC [27], and in HNSCC lymph nodes, but not at primary sites [32]; and tumor grade correlates with HSP27 [31,32,34]. A HSP27 role in HNSCC progression has been reported by some authors who indicated that HSP27 overexpression could be attributed to the overexpression of HER receptors, c-Myc, and the loss of the p53 protein [31,32].

HSP27 plays a role in the angiogenesis of many solid tumors, including HNSCC [28]. It facilitates angiogenesis by triggering calcium entry via TLR3 and activating NF-κB in human endothelial cells, leading to the release of the vascular endothelial growth factor (VEGF), a primary controller of tumor neoangiogenesis [1,24,35]. This also results in the enhancement of vascular endothelial growth factor receptor type 2 (VEGFR2) and the activation of interleukin-8 (IL-8), both of which are proangiogenic factors in the endothelium of blood vessels [1,24,35]. In breast cancer cells, HSP27 upregulates VEGF gene transcription and stimulates VEGFR2, promoting angiogenesis and cell migration [6].

### 3.2. HSP70

The HSP70 family of 13 proteins play cytoprotective and anti-apoptotic roles in cell differentiation, gene expression, immune system control, cellular senescence, and programmed cell death [1,22], and protects cells from a variety of stressors such as ischemia, infection, inflammation, heavy metals, and hyperthermia [22]. HSP70 overexpression might cause cell transformation by suppressing oncogene-induced p53-dependent and independent senescence as well as blocking myc-induced apoptosis [1]. HSP70 may enhance tumor development through the immune escape mechanism [36]. HSP70 overexpression has been reported in experimental models to enhance the tumorigenicity of transformed cells; its downregulation reduced tumorigenicity [37]. HSP70 induces carcinogenesis by stabilizing cyclin D1 expression, decreasing p53 protein, and suppressing apoptosis [1,36]. HSP70 may suppress p53 and allow the proliferation of cells with the upregulation of different oncogenes [38]. Oncogenes of the RAS signaling pathway activate two parallel pathways, including proliferation response and p53 pathway-dependent inhibition [38].

HSP70 inhibits intrinsic and extrinsic apoptosis pathways [39]. In the intrinsic mechanism, it inhibits the activation of Bax, which stabilizes the permeability of the mitochondrial membrane and protects it from the activity of factors inducing apoptosis [22]. When the extrinsic mechanism is present, HSP70 inhibits death-inducing signaling complex (DISC) [24]. Like HSP27, it is involved in caspase-dependent and caspase-independent mechanisms of apoptosis, inhibiting cell death by cathepsin modulation [1,36]. HSP70 blocks the apoptosis of cancer cells by the inhibition of caspases-3/9 activation and apoptosome formation [1]. The overexpression of HSP70 protects carcinoma cells from apoptosis by increasing the intracellular expression of the anti-apoptotic BCL-2 protein and decreasing the expression of cytochrome c [1,36,40]. HSP70 also facilitates apoptosis evasion by inhibiting PI3K/AKT signaling and initiating downstream AKT/mTOR signaling [1].

HSP70 is involved in EMT regulation [41]. Mortalin mitochondria-resident HSP70 isoforms activate EMT in different tumors, facilitating tumor invasion and metastasis [42]. Active HSP70 protein mediates invasion and EMT and comprises assisting HSP90-dependent activation of MMP-2-enhanced cell migration and metastasis [42]. The inhibition of HSP70 function reduces MMP-2 activity in cell lines and suppresses invasiveness [42].

HSP70 stabilizes the transcription factor H1F-1α, the main regulator of cancer cell hypoxia, and regulates angiogenesis [43]. A human umbilical vein endothelial cell (HUVEC) in vitro model noted that the IL-5 angiogenic activator is regulated by HSP70. An HSP70 knockdown led to the dysfunction of IL-5-induced cell proliferation, colony formation, and migration [44]. HSP70 promotes neovascularization via HIF-1/VEGF. Like VEGF, IL-5 induced HUVEC migration, colony formation in vitro, and microvessel formation in vivo [45,46]. HSP70 has a role in neoangiogenesis by the activation of HIF-1/VEGF by cancer cells and the activation of stromal endothelial cells with IL-5 help [44,45,46].

### 3.3. HSP90

As it interacts with ~400 client proteins (such as transcription factors and kinases), HSP90 has been studied in carcinogenesis [5,6,21,47]. HSP90-dependent proteins are divided into protein kinases (e.g., c-SRC/v-SRC, c RAF-1/v-RAF-1, HER2, EGFR, AKT, BRAF, MOK, and MET), transcription factors (e.g., p53 and HIF-1), and other proteins (e.g., hTERT) involved in the regulation of numerous cellular processes [5,6,19,48,49]. Some are crucial oncoproteins responsible for tumor cell survival and progression [5,6,12]. HSP90 is also involved in the evasion of apoptosis, immortalization, angiogenesis, and therapy resistance [1,19,21]. The interaction between HSP90 and components of the PI3K/AKT/mTOR signaling pathway suggests a role in the regulation of tumor cell survival [1,19]. The regulation of HIF and human telomerase reverse transcriptase (hTERT) activity by HSP90 suggests that it might have a role in tumorigenesis [19]. HSP90 may induce the immortalization of cancer cells due to the interaction with the hTERT promoter, increasing the level of telomerase in cancer cells [19]. The mechanisms through which HSP90 exerts its anti-apoptotic effects include a reduction in the activity of caspase-3 and caspase-8, a decrease in the quantity of tumor necrosis factor and FAS receptors, alterations in the levels of transcription factors p53 and NF-κB, and an imbalance in the pro- and anti-apoptotic proteins of the Bc1-2 family, favoring the latter [49,50]. This dominance of anti-apoptotic protein expression inhibits a decrease in the mitochondrial transmembrane potential [48,49]. HSP90 can block apoptosis by connecting with APAF-1, which inhibits the oligomerization of APAF-1 mediated by cytochrome c, and activates procaspase-9 [48]. HSP90 also blocks apoptosis through the formation of a triple complex with the pro-apoptotic kinase ASK1 and AKT. HSP 90 clients, such as AKT, may promote the anti-apoptotic influence of tumor cells through the downregulation of members belonging to the apoptotic machinery [19,48]. HSP90 also inhibits intracellular transport of the apoptosis-inducing factor (AIF) and endonuclease G from the mitochondrion to cytosol [51,52]. HSP90 inhibits apoptosis by p53 protein stabilization in both wild-type and mutated p53, protecting it from proteasome degradation [49].

HSP90 binds with some of cytoskeletal proteins, such as NCK-associated protein 1 (NCKAP1) and Wiskott–Aldrich syndrome protein family member 3 (WASF3), which are involved in the regulation of actin polymerization and are responsible for cell motility [53]. The mechanism of metastasis in various tumors, including HNSCC, is induced by the cooperation of extracellular HSP90 with MMP-2 and MMP-9, which disrupts the extracellular matrix and leads to the dissemination of tumor cells and formation of metastasis [2,54]. HSP90 is secreted by tumor cells, interacting with MMP-2 and MMP-9 [49]. The direct activation of MMP-2 by HSP90 increases tumor cell motility and invasiveness [2,5,54]. HSP90 engages with various receptors (including HER2, EGFR, and LPR1) to facilitate downstream signaling pathways related to tumor proliferation and metastasis, resembling the EMT phenotype [49].

HSP90 expression in OSCC is associated with nodal metastasis and an advanced tumor stage, suggesting that HSP90 may play a role in tumor progression. This may reflect HSP90 interaction with the NF-ĸB and PI3K/AKT pathways [6,12,47,51]. The association between HSP90 and AKT and hypoxia-inducible factor 1-alpha (HIF-1α) contributes to the functional stabilization of PI3K/AKT signaling vital for glycolysis-dependent tumor cell survival and participates in the control of metabolic reprograming [2] that enables tumor cells to sustain elevated proliferation rates and endure stressors. Radiotherapy may change the metabolism of tumor cells, leading to alterations in levels of metabolites, inducing oxidative stress and increasing the production of reactive oxygen species (ROS) that modify the network of cellular signaling pathways responsible for cell survival and apoptosis [6,12,52].

## 4. The Clinicopathological and Prognostic Significance of HSPs in HNSCC

The prognostic value of HSPs in HNSCC is unclear, although there are some correlations that medium and large HSPs correlate with more advanced disease and worse overall survival (OS). Previous clinical studies have been limited, usually single-center with fewer than 100 cases, have not distinguished HPV+ from HPV- tumors, and have used heterogenous methods, although they have usually used immunohistochemistry. Table 1 summarizes the significant (*p* < 0.05) clinicopathological findings for each HSP.

The most controversial findings are related to HSP 27, where some studies show that HSP27 overexpression is correlated with a lower pT stage, lower tumor grade, better prognosis, and longer OS [55,56,57,58,59,60,61]. In contrast, other studies suggest that HSP27 may be responsible for tumor growth and progression. Its increased expression was associated with more severe dysplasia (suggesting the role of HSP27 in EMT and tumor initiation) [34], a higher tumor grade [62], higher pT stage [59], higher clinical stage [63], lymph node metastasis [59,64], and worse OS [64].

HSP47 expression in HNSCC has yielded inconsistent findings. Its overexpression was reported to correlate with better OS [65] and with worse OS [66,67].

Only a few studies have reported on HSP60 in HNSCC. Worse OS, as well as a higher clinical stage, poor prognosis, and the presence of nodal metastasis have been reported to be associated with HSP60 overexpression [66,68].

Several studies have reported correlations between HSP70 and advanced HNSCC, including a higher tumor grade and stage, nodal metastasis, worse OS, and shorter DFS [22,40,69,70,71,72], although one study reported better OS [59].

HSP90 overexpression in HNSCC is associated with a worse stage, lymph node metastasis, tumor progression [12,73,74,75], and worse OS [50,66,74,75,76]. Multivariate Cox regression showed that HSP90 is a poor prognostic biomarker in HNSCC [73].

Similar observations were noted for HSP105 in HNSCC. Increased HSP105 expression is correlated with an advanced clinical stage [61], poor prognosis [77], and worse OS [50,66,77].

One report showed that HSP110 overexpression is associated with worse OS in patients with HNSCC [66].

In summary, although clinical studies have been limited, most studies that show consistency suggest that the overexpression of medium and large HSPs (HSP60, HSP70, HSP90, HSP105, and HSP110) is significantly associated with worse patient OS and related factors, such as nodal status and advanced stage [12,22,50,61,66,67,68,69,71,72,73,74,76,77]. An opposite association was observed for sHSPs, such as HSP27, where its increased expression was correlated with better survival [55,56,57,58]. Similar observations were reported for other clinicopathological features of HNSCC.

**Table 1:** The summary of studies evaluating HSP overexpression and clinicopathological findings in head and neck squamous cell carcinoma.

HSP	Findings *	Method	Sample	Source
HSP27	Tumor site, lower pT stage	IHC	50	Gandour-Edwards et al., 1998 [60]
	Better survival	IHC	40	Mese et al., 2002 [55]
	Better survival, lower tumor grade, older age	IHC, WB	79	Muzio et al., 2004 [56]
	Better OS, better prognosis	IHC	57	Muzio et al., 2006 [57]
	Better OS, better prognosis, lower tumor grade	IHC	80	Wang et al., 2009 [58]
	Early clinical stage, lower tumor grade	IHC	56	Mohtasham et al., 2011 [61]
	Worse OS, lymph node metastasis	IHC	50	Kaigorodova et al., 2016 [64]
	Higher clinical stage	IHC, PCR	44	Karam et al., 2017 [63]
	Higher tumor grade	IHC	30	Ajalyakeen et al., 2020 [62]
	Better OS, better prognosis, older age, higher pT stage, lymph node metastasis	IHC/TCGA	158/112	Borowczak et al., 2025 [59]
HSP47	Better OS, lower tumor grade	IHC, WB	50	Song et al., 2017 [65]
	Worse OS	TCGA	504	Fan et al., 2020 [66]
	Worse survival, shorter DFS	IHC	339	Da Costa et al., 2023 [67]
HSP60	Worse OS	TCGA	504	Fan et al., 2020 [66]
	Worse OS, poor prognosis, higher clinical stage, lymph node metastasis	IHC	79	Zhou et al., 2023 [68]
HSP70	Tumor size, tumor grade	IHC, WB	38	Kaur et al., 1995 [78]
	Lower pT stage	IHC	50	Gandour-Edwards et al., 1998 [60]
	Shorter DFS, higher tumor grade, shorter transition time	IHC	125	Kaur et al., 1998 [69]
	Higher tumor grade, no lymph node metastasis	IHC	41	Lee et al., 2008 [70]
	Shorter DFS, tumor location, lymph node metastasis, tumor grade	IHC	90	Choi et al., 2015 [71]
	Lower clinical stage, no lymph node metastasis, smaller tumor size	IHC	50	Taghavi et al., 2018 [79]
	Higher tumor grade	IHC	15	Priyanka et al., 2019 [40]
	Older age	IHC	117	Venugopal et al., 2022 [80]
	Worse survival, poor prognosis, higher clinical stage, pT stage	IHC	104	Ceylan et al., 2022 [72]
	Higher TNM stage, higher tumor grade, lymph node metastasis, higher recurrence rate, shorter DFS, larger tumor size	IHC	50	Elhendawy et al., 2023 [22]
	Better OS, higher tumor grade	IHC/TCGA	158/112	Borowczak et al., 2025 [59]
HSP90	Lymph node metastasis, worse survival	IHC	36	Chang et al., 2017 [74]
	Worse survival	WB	499	Ono et al., 2018 [50]
	Worse OS	TCGA	504	Fan et al., 2020 [66]
	Lymph node metastasis	IHC	58	Shiraishi et al., 2021 [12]
	Worse survival	IHC/TCGA	97/98	Santos et al., 2021 [76]
	Higher pT stage, survival status, poor prognosis	IHC	56	Bar et al., 2021 [73]
	Worse OS, higher pT stage, lymph node metastasis	IHC/TCGA	68/499	Zhang et al., 2022 [75]
	Worse OS, higher pT stage, clinical stage, lymph node metastasis	IHC, WB, PCR/TCGA	59/419	Tang et al., 2023 [20]
HSP105	Advanced clinical stage	IHC	56	Mohtasham et al., 2011 [61]
	Worse survival	WB	499	Ono et al., 2018 [50]
	Worse OS	TCGA	504	Fan et al., 2020 [66]
	Worse survival, poor prognosis	IHC	70	Arvanitidou et al., 2020 [77]
HSP110	Worse OS	TCGA	504	Fan et al., 2020 [66]

Abbreviations: OS—overall survival, DFS—disease-free survival, IHC—immunohistochemistry, WB—Western blot, TCGA—The Cancer Genome Atlas, PCR—polymerase chain reaction. * This table presents only statistically significant correlations found in the cited studies (*p* < 0.05).

## 5. HSPs and Cancer Stem Cells

Cancer stem cells (CSCs) in HNSCC and their role in tumorigenesis, tumor progression, metastasis, tumor recurrence, and resistance to therapy are well-discussed, including their origin and facilitation by EMT [81,82,83,84]. Various genetic and epigenetic factors regulate their biological behavior, such as transcription factors, signaling pathways, and molecular chaperones [82,85]. The roles of transcription factors and signaling pathways are also well-discussed. The role of HSPs in CSCs is unknown [82,85].

### 5.1. HSP27

HSP27 is involved in tumorigenesis and the resistance of the tumor to chemotherapy and radiotherapy [86]. Because the phosphorylation of HSP27 facilitates the ubiquitination and proteasomal breakdown of proteins that drive stemness (including NANOG, OCT4, c-Myc, SOX2, and KLF4) in non-small-cell lung cancer (NSCLC) cells, the inactivation of p38 promotes the expression of CSC characteristics [87]. Phosphorylated HSP27 may act in various functions in cell biology, depending on the specific tumor [87].

Colorectal CD133+ CSCs with HSP27 expression prevent caspase-9 and caspase-3 cleavage in the cell death cascade, and the inhibition of HSP27 may enhance apoptosis in response to hypoxia [88]. Phosphorylation of HSP27 is enhanced in ALDH1+ breast cancer stem cells and is required for the functioning of CSCs in different cancers [1]. Experimental findings indicate that HSP27 and its phosphorylation play a role in the vasculogenic activity induced by EGF in breast cancer stem/progenitor cells [1,89]. Phosphorylated HSP27 induces EMT and activates NF-ĸB, thereby taking part in the maintenance of stem cells [46].

Oral CSC-like cells with increased HSP27 expression are associated with resistance to apoptosis, oxidative stress, and cytotoxic drugs [90]. Phosphorylated HSP27 appeared to provide protection against caspase-dependent apoptosis triggered by a lack of oxygen or serum depletion in CD133+ CSCs derived from various tumor, including OSCC [82,88]. This mechanism was linked to the p38 MAPK/MAPKAPK2/HSP27 pathway, which is inhibited by the protein phosphatase PP2A which is responsible for the dephosphorylation of HSP27 [82,88]. There is a report showing that the p38/HSP27 pathway enhances EMT induction in oral cancer cells [90].

### 5.2. HSP70

Increased expression of HSP70 in CSC-like cells has been reported in medulloblastoma and gastric and breast cancer [91,92,93]. HSP70 expression in breast cancer cells is associated with the expression of stem cell markers (CD44 and Sca1) and the high metastatic potential of tumor cells [92]. EMT is an important feature of CSCs, which determines their biological behavior. HSP70 plays a role in cancer stemness by increasing the expression of cancer stemness-associated proteins (such as vimentin, N-cadherin, MMP-2, MMP-9, Snail, Slug, Twist, and others) [94]. HSP70 is also associated with high CSC resistance to apoptosis [82]. HSP70 is expressed in the hypoxic regions of tumor tissue where EMT is present, indicating an association between HSP70 and the development and maintenance of cancer stemness [82]. GRP75 (mortalin) may contribute to EMT and cancer stemness, and correlate with the aggressive behavior of mammary gland carcinomas [82,95]. Tumor-initiating CSCs in breast cancer tissue may be a result of oncogenic mutations in normal stem cells or progenitors; increased levels of HSP70 are necessary to provide their ‘stemness’, contributing to tumor progression [82,96]. There may be a role of HSP70 in CSC resistance to therapy. An experimental study in lung and breast cancer showed that HSP chaperonage is enhanced in CSCs by the transcriptional upregulation of proteins in the HSP family, including HSP70 [97,98].

### 5.3. HSP90

HSP90 regulates the CSC phenotype and maintenance by interacting with proteins expressed by stem cells [82]. HSP90 cooperates with various transcriptional factors (including OCT4, NANOG, STAT3, and JAKs) which influence stem cell pluripotency [49]. For example, HSP90 abnormally activates EGFR, AKT, Hedgehog, WNT/β catenin, and Src, components of signaling pathways which promote the self-renewal of CSCs in malignant tissue [82,99,100]. WNT and Hedgehog signaling are strongly activated in HNSCC and are associated with CD133, SOX2, and CXCR4 expression, and most of the analyzed HNSCCs presented high expressions of HSP90 [73,101,102]. The direct interaction between HSP90 function and BMI1 gene transcription expression and CSC self-renewal in HNSCC was reported in one study [103]. HSP90 was reported to promote EMT via the activation of HIF1α and NF-қB and the enhancement of CSC accumulation [99,100]. The inhibition of HSP90 by Ku711 and Ku757 in HNSCC decreased stem cell markers (such as CD44 and ALDH) but increased E-cadherin [103]. Targeted therapy with HSP90 inhibitors may lead to a reduction in the population of ALDH+/CD44+ cells and downregulation of certain microRNAs related to the resistance of CSCs to chemotherapy [103]. Data on HSP90 chaperone function concerning activation of the AKT/MEK/ERK/JAK2/STAT3 signaling network in triple-negative breast cancer showed the generation of CSC-like cells, which demonstrated CD44 expression and high ALDH1 activity [104]. It has been proposed that HSP90 activates CSC mechanisms which upregulate the expression of MMP-2 and MMP-9 and that interaction with secreted MMPs induces local degradation of the extracellular matrix and enhances tumor cells migration, especially those with the CSC phenotype [82]. This is rational because MMPs increase the degradation of selected immunoreactive proteins at the surface of CSCs, thereby making CSCs less visible to the immunological system [82]. Many reports have demonstrated that molecular chaperones are determinants of cancer stemness development, which define the formation of the CSC phenotype and mediate the tumorigenic properties of CSCs [82,90,91]. Most CSC features are linked to the activity of molecular chaperones (especially HSP27, HSP70, and HSP90), which determine the migration, invasion, and resistance to radiotherapy and chemotherapy in solid tumors, including HNSCC [1,49,82,90,92,103].

## 6. HSP Inhibitors

HSPs are targetable as anticancer therapy. HSP inhibitors have been explored in treating some malignancies, including breast, lung, pancreatic, and gastrointestinal cancers, and melanoma [6,12,19,24,86,105,106]. However, studies on HNSCC are limited. HSP inhibitors are classified according to the specific HSP targeted: HSP27, HSP70, or HSP90. Frequently tested HSP inhibitors, potentially useful for future HNSCC therapy, are listed below:

(1) HSP27 inhibitors: quercetin, RP101 (brivudine), PA11, PA50, OGX-427 (apatorsen), and ivermectin.
(2) HSP70 inhibitors: apoptozole, triptolide, minnelide, artesunate, PES (pifithrin-μ), MKT-077, YM-01, and YM-08.
(3) HSP90 inhibitors: geldanamycin, 17-AAG (tanespimycin), 17-DMAG (alvespimycin), IPI-504 (retaspimycin), IP-493, WK881, radicicol, NVP-AUY922 (luminespib), AT13387 (onalespib), GRP94 (glucose-regulated protein 94), STA-9090 (ganetespib), CNF-2024 (BIIB021), CUDC-305 (Debio 0932), PU-H71 (zelavespib), XL888, NVP-BEP80, sansalvamide, novobiocin, TAS-116 (pimitespib), HS-196, SNX-5422, Ku363, Ku711, and Ku757.

### 6.1. HSP27 Inhibitors

Three approaches have been investigated for targeting HSP27 in cancer cells [6,24,86]:

(1) Small-molecule inhibitors—quercetin and RP101—which are under in vitro and in vivo evaluation; they increase anticancer impact in several cancer cell lines, including HNSCC [24,86,105,107,108].
(2) Peptide aptamers—PA11 and PA50.
(3) Antisense oligonucleotides—such as OGX-427 (apatorsen)—which interact directly with HSP27 have been investigated.

The most-studied HSP27 inhibitor is quercetin, a bioflavonoid with anticancer properties that targets heat shock transcriptional factor 1-related HSPs in many tumor cell lines [6,24,86,109]. There are several in vitro studies evaluating its use in HNSCC, especially in OSCC. It was reported that quercetin significantly inhibits cell proliferation and induces apoptosis in OSCC cell lines [110,111]. The mechanism of quercetin activity is complex and not yet fully understood. Studies have focused on the mechanisms underlying its anticancer effects, which may include the following:

(a) The inhibition of cell invasion, migration, and colony formation by suppressing MMP-2 and MMP-9 activity [112].
(b) The inhibition of the progression of OSCC by the activation of the miR- 1254/CD36 pathway [113].
(c) The inhibition of glycolysis and cell proliferation of OSCC cells by suppressing the G3BP1/YWHAZ 919 axis [110].
(d) Ferroptosis (programmed cell death characterized by iron dependency] may also be a mechanism [114].
(e) The provision of an unfavorable microenvironment for HPV+ HNSCC by increasing reactive oxygen species production, decreasing tumor pH, and inhibiting tumor growth [115].

Quercetin provides synergy with chemotherapy in HNSCC. Quercetin with cisplatin or gemcitabine increases the chemosensitivity of oral tumor cells [109]. A similar effect was observed when quercetin was used with erlotinib (EGFR tyrosine kinase inhibitor) [116]. HNSCC often overexpresses EGFR, but resistance to anti-EGFR drugs develops, limiting the clinical efficacy of erlotinib. The addition of quercetin may block the development of resistance in HNSCC cells and enhance erlotinib efficacy in HNSCC [116]. However, quercetin use is limited by its low water solubility, instability under physiological conditions, and poor bioavailability [117]. Some researchers have proposed the use of photodynamic therapy to increase its bioavailability [117].

RP101 is a nucleoside unstudied in HNSCC, which may inhibit the function of HSP27 [105,118]. When it is bound to HSP27, the connection between HSP27 and AKT1, procaspase-3, and cytochrome c is reduced, impacting apoptosis [24]. RP101 is more effective when used in combination with other cytotoxic drugs. An in vivo study on rats with AH13r sarcoma cells showed that using it with cisplatin or cyclophosphamide had better antiproliferative efficacy compared to monotherapy [118].

Protein aptamers (such as PA11 and PA50) may impact HSP27 activity, increasing apoptosis in tumor cells [86]. PA11 and PA50 are negative mediators of HSP27 activity, binding to and disrupting the dimerization and oligomerization of HSP27, thereby suppressing its anti-apoptotic effect [119]. These peptide aptamers inhibit the growth of HNSCC tumors in vivo through cell cycle arrest [6,24,109,120]. They are ineffective as a monotherapy [24,86,119]; similar to small-molecule inhibitors of HSP27, protein aptamers demonstrate better efficacy when combined with other anticancer agents [86].

OGX-427 (apatorsen) is an antisense oligonucleotide. It inhibits HSP27 expression by increasing the apoptosis of tumor cells [6,121]. Combining it with conventional chemotherapy (eg gemcitabine and docetaxel) showed strong therapeutic activity, as well as with other HSP inhibitors [121,122]. The inhibition of HSP27 leads to better sensitization of NSCLC cells to erlotinib and standard chemotherapy [123]. Treatment of the multiple myeloma U266 cell line with OGX-427 or bortezomib decreased cell proliferation, enhanced apoptosis, and reduced HSP27 expression [124]. It was also found that HSP27 inhibition decreased BCL-2 expression and increased Bax expression [124]. Using OGX-427 as a radiosensitizer led to decreased neoangiogenesis and the inactivation of the AKT pathway [86]. HSP27 inhibition was also observed in the radioresistant HNSCC cell line SQ20B [125] and increased the cytotoxic effects of radiation on HNSCC cells [125].

Ivermectin is an antiparasitic drug [29]. Studies have reported that its use as an HSP27 inhibitor that may improve the targeting of oncogenes in cancer models [126]. It blocks the phosphorylation of HSP27 mediated by MAPKAPK2, disturbs the inhibition of SHPTP1 mediated by HSP27, and decreases the resistance to erlotinib [126]. It inhibited the growth of ESCC cells in culture. One study reported that it induced apoptosis in ESCC [127], while another reported that it suppressed tumor growth, esophageal metastasis, and improved chemosensitivity to cisplatin and 5-fluorouracil [128].

There is one active clinical trial evaluating HSP27 inhibitors in HNSCC (NCT05724329): quercetin use in combination with tislelizumab (anti-PD-1 antibody) and dasatinib in patients prior to tumor resection. In 2011, RP101 was noted to extend survival in pancreatic cancer patients in a phase II trial when used with gemcitabine. OGX-427 results were reported between 2016 and 2018 for patients with bladder, urothelial, prostate, and breast cancers [122,129,130].

### 6.2. HSP70 Inhibitors

HSP70 inhibitors may be classified into three elementary categories according to the target of binding: C-terminal peptide or substrate-binding domain (SBD), N-terminal ATP-binding domain (ABD), and HSP70 co-chaperones [2,24,131].

Apoptozole inhibits the ATPase activity of HSP70 by interacting with the ATP-binding site, inhibiting tumor cell proliferation and migration, and inducing cell apoptosis, with resultant decreased tumor growth in a xenograft model [131]. It promotes caspase-dependent apoptosis by inhibiting the connection between HSP70 and APAF-1, induces lysosome-mediated apoptosis, and decreases autophagy [131,132,133]. Its toxicity has been reported in a variety of tumor cells, including OSCC and breast and liver cancer [132,133].

Triptolide also decreases HSP70 expression. It induces apoptosis in HNSCC cell lines and inhibits the growth of tumors [134]. It inhibits the growth, invasion, migration, and angiogenesis of OSCC cells. It increases the radiosensitivity of nasopharyngeal cancer cells by inhibiting HSPA5 and inducing apoptosis [135]. It also inhibits the growth of OSCCs in tumor xenografts by suppressing the PD-L1 pathway [136]. Minnelide is a triptolide derivative that inhibits HSP70 activity. It reduces tumor growth in xenograft and metastasis models as well as reduces the expression of pro-survival proteins (e.g., c-Myc and survivin) and targets the NF-κB signaling pathway [137,138]. Its antitumor activity was reported in 2014 in two HPV+ HNSCC cell lines, inducing apoptosis and inhibiting tumor progression [139].

Artesunate is another inhibitor that decreases the expression and activity of HSP70 and has been reported to have an antitumor effect against HNSCC cells [140]. A study using cell lines showed an inhibitory effect on the growth and proliferation of HNSCC cells, causing cell cycle arrest [141]. Its cytotoxic effect was increased when used with cisplatin, and decreased levels of Rb and p-Rb were reported (directing the cell cycle to the G1/S phase, which is considered more sensitive for cisplatin) [141].

MKT-077 is an inhibitor obtained from rhodacyanine dye that impacts the interaction of nucleotide exchange factors (NEFs) with HSPA8 and HSPA9, inhibiting tumor growth and inducing cell senescence [142]. It inhibited tumor progression by releasing wild-type p53 from the HSP70–p53 complex and decreased tau protein levels in the cells [142]. The efficacy of derivatives of MKT-077, such as YM-01 and YM-08, have also been studied—YM-01 inhibited breast tumor and melanoma growth in xenograft models, and YM-08 reduced anti-tau activity in cancer cells [143].

Pifithrin-µ (PES) inhibits HSP70 by interacting with its substrate-binding domain [24]. In an animal model, PES was observed to inhibit HSP70 (HSPA1A/B)–client interaction, thereby suppressing tumor cell proliferation and migration, inducing apoptosis and cell cycle arrest, inhibiting tumor growth, downregulating the phosphorylation of AKT and ERK, dysregulating autophagy, and impairing lysosomal function. The potential of combining HSP70 inhibitors with radiotherapy, chemotherapy, or molecular-targeted therapy have occasionally been studied [135,144,145]. The combination of PES with oxaliplatin and cisplatin showed enhanced therapeutical responses in colorectal and cervical cancers [144,146].

The inhibition of HSP70 has been analyzed in clinical trials for various tumors; however, no clinical studies have been performed on HNSCC patients [145,147,148].

### 6.3. HSP90 Inhibitors

HSP90 is the most-studied target for HSP inhibitors. The first generation of HSP90 inhibitors was developed based on two potent natural inhibitors (geldanamycin and radicicol) with various subsequent derivatives (17-AAG, 17 DMAG, IPI-504, and WK881) [130,149]. A second generation of HSP90 inhibitors was derived from radicicol-based derivatives (e.g., luminespib, onalespib, glucose-regulated protein 94, and ganetespib) and purine or purine-like analogs that use X-ray crystallography to block HSP90 (e.g., BIIB021, Debio 0932, and zelavespib) [149].

Geldanamycin is the first HSP90 inhibitor. Its analogs (17-AAG, 17-DMAG, IPI-504, and IPI-493) are safer and better tolerated in clinical trials [106,130,149,150]. Geldanamycin and 17-AAG were tested in vitro on HNSCC cell lines and presented a suppressive function on tumor growth [151,152]. 17-AAG has been used in combination with radiotherapy as it enhances the sensitivity of OSCC cells to irradiation, thereby increasing the overall cytotoxic effect of therapy [153]. 17-AAG also has a synergistic effect when combined with cisplatin and induced apoptosis in cisplatin-resistant ESCC cell lines [154]. 17-AAG and retaspimycin hydrochloride (IPI-504) were also reported to be effective in glioma cell lines, where the suppression of tumor growth in immunocompromised mice was documented [106,155]. In the melanoma cell line, geldanamycin derivatives, such as 17-AAG and 17-DMAG, induced apoptosis by the activation of poly-ADP ribose polymerase (PARP) and several caspases (caspase-2, caspase-7, and caspase-9) [156]. Preclinical findings from various tumor cell lines and tumor xenograft models showed that HSP90 inhibitors, such as 17-AAG, are synergistic with taxanes in targeting NSCLC and breast cancer [19]. 17-DMAG and ganetespib reduce the viability and migration of OSCC cells [12]. Ganetespib inhibits glycolytic flux in HNSCC cells, resulting in metabolic failure and better antitumor effects [53]. It also enhances the cytotoxic effects of radiotherapy on HNSCC cells [53]. In myeloid cell lines (MCL-1) exposed to ganetespib, low expressions of anti-apoptotic proteins such as surviving and BCL-2 were observed, suggesting an apoptotic effect [157]. Ku363 is one of the novel HSP90 inhibitors that showed good in vivo efficacy in HNSCC therapy with lower toxicity than cisplatin [152]. Onalespib (AT13387) demonstrated radiosensitization in HNSCC both in vitro and in vivo [158]. It inhibits radiation-induced vasculogenesis, a typical process involved in tumor recurrence [158]. The radiosensitive effect of low doses of onalespib was confirmed in other studies on HNSCC xenografts [159,160]. Luminespib (NVP-AUY922) with cisplatin and radiotherapy was noted to be synergistic in HNSCC [161] as well as BIIB021, another HSP90 inhibitor [162]. BIIB021 demonstrated a strong antitumor effect by increasing apoptosis and enhancing cell cycle arrest [162]. A similar effect of combined therapy with HSP90 inhibition was observed with SNX5422, which increased cytotoxicity, chemosensitivity, and radiosensitivity in HNSCC [163].

NVP-BEP800 is a novel, synthetic, orally bioavailable N-terminal HSP90 inhibitor with potential selective antineoplastic activity in cancer cell lines [164]. Sansalvamide binds to N-terminal fragments of the middle domain of HSP90. These agents inhibited proliferation in melanoma cell lines [106]. Novobiocin is an aminocoumarin antibiotic that interacts with the C-terminal domain of HSP90 [130,164]. Analogs of novobiocin exhibit significant anti-proliferation influence on prostate cancer cell lines [165]. Novobiocin triggered the degradation of HSP90 client proteins such as CRAF, HER2, mutated p53, and SRC [164]. In vitro and in vivo studies demonstrated that HSP90 inhibitors suppress EMT and reduce cancer stem cells (CSCs) [82]. The inhibition of intracellular HSP90 activity by geldanamycin, 17-AAG, and 17-DMAG suppressed the proliferation and growth of CSCs [106,111,166,167]. HSP90 inhibitors can inhibit EMT together with EMT-evoked CSC accumulation, and may reverse the stemness of cancer cells by MET promotion [82]. CSCs in HNSCC are decreased by two novel HSP90 inhibitors, Ku711 and Ku757 [103]. An analysis of the signaling pathways demonstrated that these novel HSP90 inhibitors inhibit CSC pathways involved in migration, invasion, and proliferation [103].

In summary, novel HSP90 inhibitors are able to target CSCs in HNSCC and overcome resistance to radio/chemotherapy. There are several clinical trials using HSP90 inhibitors in breast, lung, and prostate cancer, melanoma, and GIST [106,168,169,170,171]. Only sporadic clinical trials have focused on HNSCC. Ganetespib was piloted in a study terminated (by the sponsor because of funding) in 2016 after four patients had received it prior to tumor resection [53]. A small phase I trial from 2015 to 2020 for patients with locoregionally advanced HNSCC used onalespib combined with cisplatin and radiotherapy (NCT02381535); however, the results are unpublished.

## 7. Summary

This is a comprehensive review of the HSP family, with particular attention to their roles in malignancy. This review includes an overview of the available information related to HNSCC. In vitro studies have shown that HSPs are involved in carcinogenesis, including tumor initiation, angiogenesis, inhibition of apoptosis, and tumor progression. HSPs are expressed in many malignancies, including HNSCC, and their expression is correlated with clinical behavior. The overexpression of many HSPs in HNSCC is associated with advanced tumors and, hence, worse survival and poor prognosis.

There are a few HSP inhibitors which have been evaluated in clinical trials not involving HNSCC, and no meaningful ones involving HNSCC. Most of the studies have not involved patients. No HSP inhibitors have been approved by the Food and Drug Administration (FDA), although pimitespib, an oral HSP90 inhibitor, was registered in Japan in 2022 for patients with GIST [172]. HSP inhibitors as a monotherapy are less useful than when used in lower doses combined with established anticancer agents or irradiation.

HSP inhibitors have potential as anticancer therapeutic agents; however, there are many reasons that attention is focused on elsewhere. Genomic alterations, epigenetic regulators, microRNAs, antibody–drug conjugates, advances in immunotherapy beyond checkpoint inhibitors, and the prospects of AI-assisted drug development are all more promising.

## Data Availability

No new data were created or analyzed in this study.
